# An Unconventional Multiple Low-Cost IMU and GPS-Integrated Kinematic Positioning and Navigation Method Based on Singer Model

**DOI:** 10.3390/s19194274

**Published:** 2019-10-02

**Authors:** Minghong Zhu, Fei Yu, Shu Xiao

**Affiliations:** College of Automation, Harbin Engineering University, Harbin 150001, China; zhuminghong@hrbeu.edu.cn

**Keywords:** unconventional, multi-sensor, kinematic trajectory model, Singer model, individual model

## Abstract

To release the strong dependence of the conventional inertial navigation mechanization on the a priori low-cost inertial measurement unit (IMU) error model, this research applies an unconventional multi-sensor integration strategy to integrate multiple low-cost IMUs and a global positioning system (GPS) for mass-market automotive applications. The unconventional integration strategy utilizes a basic three-dimensional (3D) kinematic trajectory model as the system model to directly estimate navigational parameters, and it allows the measurements from all of the sensors independently participating in measurement updates. However, the less complex kinematic model cannot realize smooth transitions between different motion statuses for the road vehicle with acceleration maneuvers. In this manuscript, we establish a more practical 3D kinematic trajectory model based on a “current” statistical Singer acceleration model to realize smooth transitions for the maneuvering vehicle. In addition, taking advantage of the unconventional strategy, we individually model the systematic errors of each IMU and the measurements of all sensors, in contrast to most existing approaches that adopt the common-mode errors for different sensors of the same design. A real dataset involving a GPS and multiple IMUs is processed to validate the success of the proposed algorithm model under the unconventional integration strategy.

## 1. Introduction

With the popularity of intelligent vehicles, positioning and navigation technology is particularly important, because the foremost requirement for all of these safety and assistance systems is an accurate knowledge of the vehicular states (including vehicle position, vehicle velocity, vehicle acceleration, vehicle attitude, etc.) at all times [1]. High-precision sensors can certainly obtain very accurate vehicular states, but their high prices prevent them from being used in general-priced vehicles. Developing affordable sensors is the main research trend for driving applications [2]. On the other hand, the sensor should offer continuous and higher-update-rate observations under all circumstances for driving applications. Thus, considering cost-efficiency and high sampling frequency, a low-cost inertial measurement unit (IMU) is a good option and could autonomously provide navigation information. Furthermore, IMUs mostly contain a gyroscope and accelerometer for all three axes which can offer sufficient navigation information about a sudden acceleration or angular and heading change [3]. However, the accuracy of sensors decreases with the price reduction, and the performance is seriously affected by accumulated bias, drift, and sensor noises [4]. While an automotive-grade gyroscope typically gives drift performance of 1°/h, a microelectromechanical system (MEMS) gyroscope has a typical performance of 70°/h [1]. Plainly, low precision is one of the most critical obstacles in the development of a low-cost IMU, which limits its applications such as navigation and guidance [5]. An approach to design sensors for systems requiring better performance than the one with a single low-accuracy IMU may offer is to fuse the measurements of multiple low-accuracy IMUs to achieve the advantage of complementarity [6]. This “wisdom of the crowd” design also has, in addition to the improved measurement performance, the benefit of making it possible for sensor fault detection and diagnosis, thereby increasing the integrity and reliability of the system [6]. Many related studies confirmed the feasibility of this design, including Leszczynski (2014), Martin (2013), Skog (2014), Wahlström (2018), etc. [6,7,8,9]. However, these studies for improving the accuracy of MEMS IMUs only focused on the device itself.

The global positioning system (GPS) is by far the most widely used high-precision localization method. However, it is further constrained by the non-availability of location during signal outage in dense urban areas [10,11]. Additionally, for determining sudden acceleration and braking, the GPS shows very limited success due to the insufficient update rate. Although both the low-cost IMU and GPS have their merits and demerits, they are complementary [4]. All things considered, one preferable approach is to integrate multiple low-cost inertial measurement units (IMUs) and one GPS receiver for kinematic positioning and navigation in mass-market automotive applications.

The optimal filter design is the key to combining multiple sensors for accuracy improvement, and the adoption of the Kalman filter (KF) is widespread. Conventionally, the Kalman filter based on integration mechanization usually adopts the inertial navigation mechanization to estimate the error states and sensor systematic errors through error measurements on the basis of the aiding sensors [12,13,14]. Its strong dependence on the a priori inertial error model may limit the use of a low-cost IMU, because the time-variant noise model could be highly sensitive to the dynamic excitations and the temperature [15]. At the same time, the direct Kalman filter, which can accurately reflect the evolution of the real state, was also occasionally discussed and applied [16,17,18]. However, these system models used in Kalman filters are all based on the a priori inertial error model. In other words, in all cases, there is no essential difference, as the IMU measurements are only applied in free inertial navigation calculation and, thus, no measurement updates are performed in Kalman filtering between adjacent measurement epochs [19]. The severe drift of low-cost IMU systematic errors occurring during GPS outages can easily result in an intolerable free inertial navigation solution between two aiding measurement updates [20,21]. To address the realization of a better utilization of measurements from IMUs in Kalman filtering, especially with low-cost IMUs, several studies were carried out. An unconventional Kalman filter was already developed by Wang and Qian [19], specifically (1) utilizing a three-dimensional (3D) kinematic trajectory model as the central system model, so that the influences of the time-variant errors of low-cost IMUs on the inertial navigation solution are essentially alleviated; (2) allowing the measurements of all sensors, IMU included, directly and independently participating in measurement updates of Kalman filtering, so that the heavy dependence of the inertial navigation mechanization on the IMU measurements in the conventional integration strategy is released [15,19]. The unconventionality of this integration strategy mainly embodies in (1) implementation of the direct estimation of the whole-value states (navigational parameters) via sensor measurements instead of the error states via error measurements; (2) the lack of the need to make a distinction between the aiding sensors and the core sensors, since the raw observables (specific forces and angular rates) of IMUs directly participate in measurement updates as the raw outputs from sensors such as a GPS does, thereby implementing true multi-sensor integration.

This research applies the unconventional integration strategy for multiple low-cost IMUs and a GPS-integrated system. There are some characteristics and some corresponding defects from previous 3D kinematic trajectory models. Inevitably, a road vehicle is certain to experience severe or light irregular changes of velocity from time to time, and the changing velocity indicates the presence of acceleration. However, the jerk vector in the kinematic trajectory model is usually treated as process noise. In most cases, the a priori knowledge of vehicle maneuvers is very little. Because the maneuvering process is controlled by human forces, it is difficult to be accurately described with mathematical formulas and can only be artificially approximated under various assumptions. Therefore, a less complex kinematic model cannot realize smooth transitions between different motion statuses for the road vehicle, which leads to an intolerable positioning and navigation solution. One should avoid employing the most complex kinematic model as the core system model throughout [18]. An appropriate model will not only be able to properly model the vehicle maneuvers, but also exhibit mathematical tractability.

We found that the key problem is how to model the unknown target accelerations. Some mature maneuvering target models were proposed on this issue. So far, the most influential maneuvering target model is the Singer model proposed by Singer in 1970, which attempts to model large-scale maneuvers by assuming a time-correlated input process and incorporating the statistics into the subsequent filter design [22]. As a global statistical model, the Singer model considers all maneuvering possibilities of targets and can be applied to many types of maneuvering. However, the Singer model is essentially an a priori model, which has some limitations in effectively describing random maneuvers of the target. Based on the Singer model, a series of modifications were developed, including the “current” statistical (CS) model, adaptive current input statistical model (ACISM), jerk model, coordinate turn model, etc. [23,24]. The common feature of these models is that the maneuver of the target is regarded as the result of a time-correlated colored noise sequence, rather than a statistically independent white noise sequence. Among them, the ACISM considers the disturbance of maneuvering acceleration caused by environmental noises and other factors, the jerk model is more suitable for describing jerky maneuvering behaviors of highly maneuvering targets (such as aircraft), and the coordinate turn model is mainly applied to turning motions. There is no comparatively great environmental noise (such as atmospheric turbulence) or strong maneuvers in the application of this research. Therefore, the “current” statistical model, which can fulfil the needs of this study, is used to model the system acceleration. As one enhanced part of this manuscript, a more practical 3D kinematic trajectory model based on the “current” statistical Singer acceleration model (CSSAM) is established as the main part of the system equation so that the smooth transitions for the maneuvering vehicle can be realized.

In addition, the significant approach to how the low-cost IMU arrays are fused in this research involves enabling the direct use of measurements for individual IMUs and separately modeling their systematic errors in Kalman filtering, in contrast to most existing approaches working under the supposition of “the common-mode errors of different sensors of the same design” [7], which means modeling their systematic errors as a group of the shared states in KF from the view of algorithm design. Usually, one created a high-performance artificial or virtual IMU to be equivalent to this IMU array [8,25]. In general, those IMU systematic errors are different from run to run and in-run even with the same IMU sensor, which was recognized and largely admitted by the community of IMU manufacturers and users. The centralized filter fusion in Bancroft and Lachapelle (2011) could process the relative position, velocity, and attitude between the IMUs in order not to repeatedly use the GPS measurements once a GPS aiding epoch became available [25]. However, none of the aforementioned outcomes allowed applying the measurements from each IMU and GPS receiver independently in KF measurement updates. As another enhanced part of this manuscript, measurements and systematic errors of these IMU arrays are individually modeled in Kalman filtering, instead of adopting a set of common shared states for all IMUs. Therefore, the effect of the noises of low-cost IMU raw outputs could be markedly reduced because both the raw data and systematic errors participate in Kalman filtering updates. Moreover, we have the choice of multiple low-cost IMUs and need not stick to the ones of the same design or from the same vendor.

As a matter of fact, given the integration strategy and the system model, various nonlinear filtering candidates (e.g., extended Kalman filter (EKF), unscented Kalman filter (UKF), particle filter (PF), etc.) are available. Nevertheless, this manuscript uses the EKF instead of other nonlinear filtering algorithms because (1) the research does not focus on the filtering techniques, (2) the system model does not present strong nonlinearity, and (3) the EKF algorithm is simple and easy to use in practical engineering.

A more practical system motion model is described in Section 2. The formulation of the unconventional multi-sensor Kalman filter is presented in Section 3. The implementation of the Kalman filter is discussed in Section 4. The data processing results from road tests demonstrate the feasibility by adopting the proposed algorithm model under the unconventional integration strategy. The corresponding numerical results along with analysis are provided in Section 5. Conclusions and remarks are given in Section 6.

## 2. The System Motion Model

The general system motion model contains three parts: (1) the 3D kinematic trajectory [26], (2) the attitudes, and (3) the angular rate.

### 2.1. Three-Dimensional Kinematic Trajectory Model

**Figure 1:**
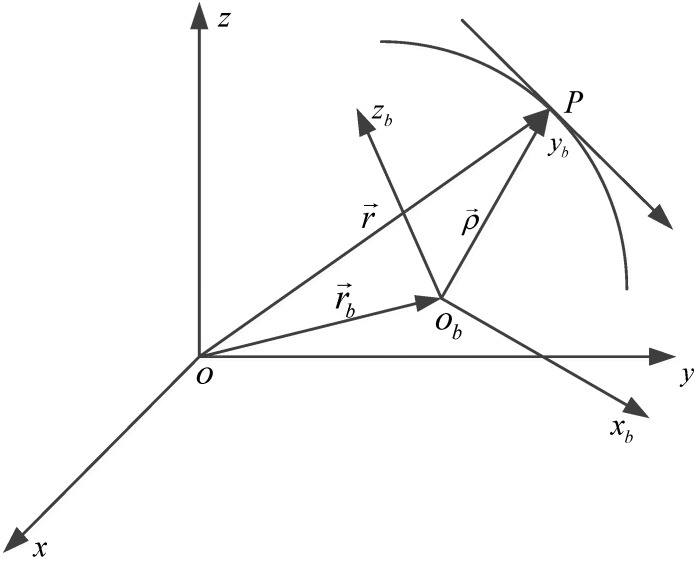
The coordinate systems.

The instantaneous motion of a rigid body can be described using kinematics without considering the forces that cause different types of motion. For a mechanical system, suitable coordinate systems are necessary to give a mathematical expression of the position, velocity, and acceleration. Two reference frames move relative to each other, as shown in Figure 1. One is the space-fixed system oxyz(S), and the other is the moving system $o_{b}x_{b}y_{b}z_{b}(S_{b})$.

In Figure 1, the time-dependent position vector $\vec{r}$ of a point P is uniquely expressed using three unit vectors $\vec{i},\vec{j},\vec{k}$ along three axes x,y,z in frame S.

(1) $$\vec{r}=x\vec{i}+y\vec{j}+z\vec{k}.$$

In components, the motion of the moving point at a time instant t with respect to a start time $t_{0}$ can be given as shown below.

(2) $$x(t)=x(t_{0})+\dot{x}(t_{0})(t-t_{0})+\frac{1}{2}\ddot{x}(t_{0})(t-t_{0}{)}^{2}+\frac{1}{6}\dddot{x}(t_{0})(t-t_{0}{)}^{3}+\cdots ,$$

(3) $$y(t)=y(t_{0})+\dot{y}(t_{0})(t-t_{0})+\frac{1}{2}\ddot{y}(t_{0})(t-t_{0}{)}^{2}+\frac{1}{6}\dddot{y}(t_{0})(t-t_{0}{)}^{3}+\cdots ,$$

(4) $$z(t)=z(t_{0})+\dot{z}(t_{0})(t-t_{0})+\frac{1}{2}\ddot{z}(t_{0})(t-t_{0}{)}^{2}+\frac{1}{6}\dddot{z}(t_{0})(t-t_{0}{)}^{3}+\cdots ,$$

where $\dot{x},\ddot{x},\dddot{x},$ $\dot{y},\ddot{y},\dddot{y},$ $\dot{z},\ddot{z},\dddot{z},\ldots$ are the first, second, and third derivatives of x,y,z with respect to t. It is noteworthy that the time interval $\left(t-t_{0}\right)$ must be short enough in order to require as few terms as possible. Let ${\vec{r}}_{0}$ be the position vector of the origin $O_{b}$, and let $\vec{\rho }$ and ${\vec{\rho }}_{b}$ be the relative position vector of the point P from $O_{b}$ and the same vector in $S_{b}$; then, we can get the following relationship:

(5) $$\vec{r}(t)={\vec{r}}_{0}(t)+\vec{\rho }(t)={\vec{r}}_{0}(t)+D^{T}(t){\vec{\rho }}_{b}(t),$$

where D(t) is the instantaneous rotation matrix from S to $S_{b}$. According to Equation (5), we can successively derive the velocity vector, acceleration vector, and jerk vector. Moreover, D(t) is coupled with the angular motion.

With respect to a rigid body in motion, the basic trajectory parameters are considered in the local navigation frame ($S_{n}$), i.e., the position $r_{nb}^{n}$ of the IMU center, the velocity $v_{nb}^{n}$, the acceleration $a_{nb}^{n}$, and the jerk $j_{nb}^{n}$. In essence, $v_{nb}^{n}$ is the derivative of $r_{nb}^{n}$, and it is also transformed from its opposite number $v_{nb}^{b}$ in the body frame ($S_{b}$). Likewise, $a_{nb}^{n}$ and $j_{nb}^{n}$ are the derivatives of $v_{nb}^{n}$ and $a_{nb}^{n}$, respectively. According to the vector dynamics, the trajectory parameters are directly derived as shown below.

(6) $${\dot{r}}_{nb}^{n}=v_{nb}^{n}=C_{b}^{n}v_{nb}^{b},$$

(7) $${\dot{v}}_{nb}^{n}=a_{nb}^{n}=C_{b}^{n}[\omega_{nb}^{b}\times ]v_{nb}^{b}+C_{b}^{n}{\left(\begin{array}{ccc} {\dot{v}}_{nbx}^{b} & {\dot{v}}_{nby}^{b} & {\dot{v}}_{nbz}^{b} \end{array}\right)}^{T}=C_{b}^{n}a_{nb}^{b},$$

(8) $${\dot{a}}_{nb}^{n}=j_{nb}^{n}=C_{b}^{n}[\omega_{nb}^{b}\times ]a_{nb}^{b}+C_{b}^{n}{\left(\begin{array}{ccc} {\dot{a}}_{nbx}^{b} & {\dot{a}}_{nby}^{b} & {\dot{a}}_{nbz}^{b} \end{array}\right)}^{T}=C_{b}^{n}j_{nb}^{b},$$

where $r_{nb}^{n},v_{nb}^{n},a_{nb}^{n},j_{nb}^{n},\omega_{nb}^{n}$ are the position, velocity, acceleration, jerk, and angular rate vectors in $S_{n}$, respectively. $v_{nb}^{b},a_{nb}^{b},j_{nb}^{b}$ are the velocity, acceleration, and jerk vectors in $S_{b}$. $C_{b}^{n}$ is the direction cosine matrix (DCM) from $S_{b}$ to $S_{n}$. The derivatives of the body velocity components and the body acceleration components are given below.

$$\begin{array}{c} \left[\begin{array}{c} {\dot{v}}_{nbx}^{b}\\ {\dot{v}}_{nby}^{b}\\ {\dot{v}}_{nbz}^{b} \end{array}\right]=\left[\begin{array}{c} a_{nbx}^{b}\\ a_{nby}^{b}\\ a_{nbz}^{b} \end{array}\right]-\left[\begin{array}{ccc} 0 & -\omega_{nbz}^{b} & \omega_{nby}^{b}\\ \omega_{nbz}^{b} & 0 & -\omega_{nbx}^{b}\\ -\omega_{nby}^{b} & \omega_{nbx}^{b} & 0 \end{array}\right]\left[\begin{array}{c} v_{nbx}^{b}\\ v_{nby}^{b}\\ v_{nbz}^{b} \end{array}\right],\\ \left[\begin{array}{c} {\dot{a}}_{nbx}^{b}\\ {\dot{a}}_{nby}^{b}\\ {\dot{a}}_{nbz}^{b} \end{array}\right]=\left[\begin{array}{c} j_{nbx}^{b}\\ j_{nby}^{b}\\ j_{nbz}^{b} \end{array}\right]-\left[\begin{array}{ccc} 0 & -\omega_{nbz}^{b} & \omega_{nby}^{b}\\ \omega_{nbz}^{b} & 0 & -\omega_{nbx}^{b}\\ -\omega_{nby}^{b} & \omega_{nbx}^{b} & 0 \end{array}\right]\left[\begin{array}{c} a_{nbx}^{b}\\ a_{nby}^{b}\\ a_{nbz}^{b} \end{array}\right], \end{array}$$

where $j_{nbx}^{b},j_{nby}^{b},j_{nbz}^{b}$ are the body jerk components, $\omega_{nbx}^{b},\omega_{nby}^{b},\omega_{nbz}^{b}$ are the body angular rate components, $v_{nbx}^{b},v_{nby}^{b},v_{nbz}^{b}$ are the body velocity components, and $a_{nbx}^{b},a_{nby}^{b},a_{nbz}^{b}$ are the body acceleration components.

### 2.2. “Current” Statistical Singer Acceleration Model

The Singer model assumes that the maneuvering acceleration a(t) obeys a stationary first-order time-correlated process with zero-mean. The correlation function is expressed in the form of exponential decay as

(9) $$R_{a}(\tau )=E[a(t)a(t+\tau )]=\sigma_{a}^{2}e^{-\alpha |\tau |},\alpha \geq 0,$$

where $\sigma_{a}^{2}$ is the variance of the target acceleration, and α is the reciprocal of the maneuver (acceleration) time constant, which is named “maneuvering frequency” and taken generally as an empirical value. Assuming that the probability density function of the acceleration approximately obeys uniform distribution, $\sigma_{a}^{2}=\frac{a_{\max}^{2}}{3}(1+4P_{\max}-P_{0})$ with the maximum maneuvering acceleration $a_{\max}$ and its probability $P_{\max}$, and the non-maneuvering probability $P_{0}$.

Utilizing the correlation function $R_{a}(\tau )$, the acceleration a(t) may be expressed in terms of white noise by the Wiener–Kolmogorov whitening procedure [16], as shown below.

(10) $$\dot{a}(t)=-\alpha a(t)+w_{a}(t),$$

where $w_{a}(t)$ is the white Gaussian noise with the mean of 0 and the variance of $2\alpha \sigma_{a}^{2}$.

The “current” statistical (CS) model adopts a non-zero mean and modified Rayleigh distribution to characterize the maneuvering acceleration. Specifically, the modified Rayleigh distribution is used to describe the “current” probability density of maneuvering acceleration, and the mean value is the “current” acceleration prediction. The random acceleration still conforms to the first-order time-correlated process. According to the Singer model and the CS model, the body acceleration can be given as

(11) $$\begin{array}{l} a_{nb}^{b}(t)=\bar{a}(t)+a(t)\\ \dot{a}(t)=-\alpha a(t)+w_{a}(t) \end{array},$$

where $\bar{a}(t)$ is the “current” mean of maneuvering acceleration, and it is constant during each sampling period.

The differential equation of body acceleration is acquired by consolidating Equation (11), as shown below.

(12) $${\dot{a}}_{nb}^{b}(t)=-\alpha a_{nb}^{b}(t)+\alpha \bar{a}(t)+w_{a}(t).$$

Then, the full-state differential equation in a continuous-time system is as follows:

(13) $$\left[\begin{array}{c} \int a_{nb}^{b}(t)d_{t}\\ a_{nb}^{b}(t)\\ {\dot{a}}_{nb}^{b}(t) \end{array}\right]=\left[\begin{array}{ccc} 0 & 1 & 0\\ 0 & 0 & 1\\ 0 & 0 & -\alpha \end{array}\right]\left[\begin{array}{c} \iint a_{nb}^{b}(t)d_{t}d_{t}\\ \int a_{nb}^{b}(t)d_{t}\\ a_{nb}^{b}(t) \end{array}\right]+\left[\begin{array}{c} 0\\ 0\\ \alpha \end{array}\right]\bar{a}(t)+\left[\begin{array}{c} 0\\ 0\\ 1 \end{array}\right]w_{a}(t).$$

### 2.3. Attitude Angle Model

Typically, Euler angles (i.e., pitch, roll, and heading) of $S_{b}$ with respect to $S_{n}$ are selected to demonstrate the rotating properties of a 3D object. The differential equations of Euler angles are given in matrix form as follows:

(14) $$\left[\begin{array}{c} \dot{P}\\ \dot{\gamma }\\ \dot{\psi } \end{array}\right]=\left[\begin{array}{ccc} \cos\gamma & 0 & \sin\gamma \\ \sin\gamma \tan P & 1 & -\cos\gamma \tan P\\ \sin\gamma \sec P & 0 & -\cos\gamma \sec P \end{array}\right]\left[\begin{array}{c} \omega_{nbx}^{b}\\ \omega_{nby}^{b}\\ \omega_{nbz}^{b} \end{array}\right]=C_{3\times 3}\omega_{nb}^{b},$$

wherein P,γ,ψ are the pitch, roll, and heading, respectively, $\omega_{nb}^{b}$ is the angular rate in $S_{b}$, and $\omega_{nbx}^{b},\omega_{nby}^{b},\omega_{nbz}^{b}$ are the components of $\omega_{nb}^{b}$ in three axes.

### 2.4. Angular Rate Model

For a normally running vehicle with a smooth steering within a small time interval, three components of $\omega_{nb}^{b}$ are reasonably treated as independent. In a general way, $\omega_{nbx}^{b}$ and $\omega_{nby}^{b}$ are both modeled as zero-mean processes by the first-order Markov model, and $\omega_{nbz}^{b}$ is modeled as a non-zero-mean random process with random disturbance. More concretely, the zero-mean Singer motion model is adopted to describe the dynamic variation of $\omega_{nbx}^{b}$ and $\omega_{nby}^{b}$ in the system model, and the modified Singer model is used to express the dynamics of $\omega_{nbz}^{b}$ [22,27,28].

## 3. The Formulation of the Unconventional Kalman Filter

**Figure 2:**
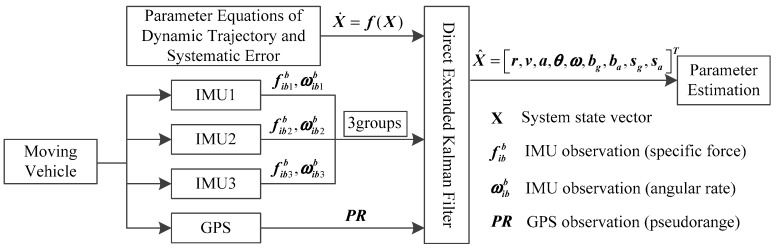
Unconventional integration mechanism.

In a general way, the state and measurement vector ought to be arranged for the system Kalman filter. In this research, the whole value state which describes the kinetic characteristic of the vehicle is one part of the state vector. The systematic errors of each IMU, e.g., the biases, scale factor errors, and so forth, are also modeled individually in the Kalman filter, instead of assuming “the common-mode errors of different sensors of the same design”. Therefore, the systematic error is the other part. As for the measurement vector, the raw observables from the GPS and multiple IMUs are considered. Figure 2 illustrates the unconventional integration mechanism in this research. To demonstrate the benefits of the individual modeling for systematic errors and measurements, choosing different low-cost IMUs from different vendors is the best choice. Considering the cost of the experiment and the amount of calculation, it is advisable to integrate three low-cost IMUs and one GPS receiver in the kinematic positioning and navigation system.

### 3.1. The State Vector Including the Systematic Errors in Kalman Filter

In this manuscript, the state vector of the constructed multi-sensor integration Kalman filter contains 51 components as follows:

$$X_{51\times 1}={\left[\begin{array}{ccccccccccccccccc} r^{T} & {\left(v_{nb}^{b}\right)}^{T} & {\left(a_{nb}^{b}\right)}^{T} & \theta^{T} & {\left(\omega_{nb}^{b}\right)}^{T} & b_{g1}^{T} & b_{g2}^{T} & b_{g3}^{T} & b_{a1}^{T} & b_{a2}^{T} & b_{a3}^{T} & S_{g1}^{T} & S_{g2}^{T} & S_{g3}^{T} & S_{a1}^{T} & S_{a2}^{T} & S_{a3}^{T} \end{array}\right]}^{T},$$

where the position vector is expressed by triaxial coordinates in the earth-fixed coordinate system $r={\left(\begin{array}{ccc} X & Y & Z \end{array}\right)}^{T}$, the body velocity vector $v_{nb}^{b}={\left(\begin{array}{ccc} v_{nbx}^{b} & v_{nby}^{b} & v_{nbz}^{b} \end{array}\right)}^{T}$, the body acceleration vector $a_{nb}^{b}={\left(\begin{array}{ccc} a_{nbx}^{b} & a_{nby}^{b} & a_{nbz}^{b} \end{array}\right)}^{T}$, the attitude $\theta ={\left(\begin{array}{ccc} P & \gamma & \psi \end{array}\right)}^{T}$, the body angular rate vector $\omega_{nb}^{b}={\left(\begin{array}{ccc} \omega_{nbx}^{b} & \omega_{nby}^{b} & \omega_{nbz}^{b} \end{array}\right)}^{T}$, the gyroscope bias vector $b_{g1}={\left(\begin{array}{ccc} b_{g1x} & b_{g1y} & b_{g1z} \end{array}\right)}^{T}$, $b_{g2}={\left(\begin{array}{ccc} b_{g2x} & b_{g2y} & b_{g2z} \end{array}\right)}^{T}$, $b_{g3}={\left(\begin{array}{ccc} b_{g3x} & b_{g3y} & b_{g3z} \end{array}\right)}^{T}$, the accelerometer bias vector $b_{a1}={\left(\begin{array}{ccc} b_{a1x} & b_{a1y} & b_{a1z} \end{array}\right)}^{T}$, $b_{a2}={\left(\begin{array}{ccc} b_{a2x} & b_{a2y} & b_{a2z} \end{array}\right)}^{T}$, $b_{a3}={\left(\begin{array}{ccc} b_{a3x} & b_{a3y} & b_{a3z} \end{array}\right)}^{T}$, the gyroscope scale factor error $s_{g1}={\left(\begin{array}{ccc} s_{g1x} & s_{g1y} & s_{g1z} \end{array}\right)}^{T}$, $s_{g2}={\left(\begin{array}{ccc} s_{g2x} & s_{g2y} & s_{g2z} \end{array}\right)}^{T}$, $s_{g3}={\left(\begin{array}{ccc} s_{g3x} & s_{g3y} & s_{g3z} \end{array}\right)}^{T}$, and the accelerometer scale factor error $s_{a1}={\left(\begin{array}{ccc} s_{a1x} & s_{a1y} & s_{a1z} \end{array}\right)}^{T}$, $s_{a2}={\left(\begin{array}{ccc} s_{a2x} & s_{a2y} & s_{a2z} \end{array}\right)}^{T}$, $s_{a3}={\left(\begin{array}{ccc} s_{a3x} & s_{a3y} & s_{a3z} \end{array}\right)}^{T}$. Herein, subscripts 1, 2, and 3 are the sequence numbers of the IMUs.

### 3.2. The Discretization of the System Model

On the basis of the differential model in Section 2, the discrete system model for KF can be summarized after omitting the higher-order terms in Taylor series expansion. It is mentionable that the position vector r of a vehicle should be in the earth-fixed coordinate frame ($S_{e}$); thus, the position vector r at epoch k+1 in $S_{e}$ is calculated concretely as follows:

- Firstly, the local coordinate increment $\Delta r_{n}$ from epoch k to k+1 in $S_{n}$ is as follows:
  (15) $$\Delta r_{n}=\Delta tC_{b(k)}^{n}v_{nb(k)}^{b}+\frac{\Delta t^{2}}{2}C_{b(k)}^{n}a_{nb(k)}^{b}+\frac{\Delta t^{3}}{6}C_{b(k)}^{n}j_{nb(k)}^{b}.$$
- Next, $\Delta r_{n}$ is transformed from $S_{n}$ to $S_{e}$ as follows:
  (16) $$\Delta r_{e}=C_{n(k)}^{e}\Delta r_{n}.$$
- Finally, we obtain the position vector r at epoch k+1 as follows:
  (17) $$r(k+1)=r(k)+\Delta r_{e}=r(k)+C_{n(k)}^{e}\Delta r_{n}.$$

Furthermore, there is a particular discussion for the discretization of the body acceleration model. The discrete state of Equation (13) can be derived as

(18) $$Y(k+1)=\tilde{A}(k)Y(k)+\tilde{B}(k)\bar{a}(k)+w_{a}(k),$$

with $\tilde{A}(k)=\left[\begin{array}{ccc} 1 & \Delta t & {\left(\alpha \Delta t-1+e^{-\alpha \Delta t}\right)/\alpha }^{2}\\ 0 & 1 & \left(1-e^{-\alpha \Delta t}\right)/\alpha \\ 0 & 0 & e^{-\alpha \Delta t} \end{array}\right],\tilde{B}(k)=\left(\left[\begin{array}{c} \Delta t^{2}/2\\ \Delta t\\ 1 \end{array}\right]-\left[\begin{array}{c} {\left(\alpha \Delta t-1+e^{-\alpha \Delta t}\right)/\alpha }^{2}\\ \left(1-e^{-\alpha \Delta t}\right)/\alpha \\ e^{-\alpha \Delta t} \end{array}\right]\right)$.

The discrete acceleration is obtained from Equation (18) as follows:

(19) $$a_{nb(k+1)}^{b}=e^{-\alpha \Delta t}a_{nb(k)}^{b}+(1-e^{-\alpha \Delta t})\bar{a}(k)+w_{a}(k),$$

where $\bar{a}$ is the “current” mean of maneuvering acceleration, which is acquired by calculating the mean value of both sides of Equation (19), as shown below.

(20) $$\bar{a}(k+1)=E\{a_{nb(k+1)}^{b}|z^{k}\}=e^{-\alpha \Delta t}E\{a_{nb(k)}^{b}|z^{k}\}+(1-e^{-\alpha \Delta t})\bar{a}(k)=e^{-\alpha \Delta t}{\hat{a}}_{nb(k)}^{b}+(1-e^{-\alpha \Delta t})\bar{a}(k),$$

where $z^{k}$ is the sequence of all measurements up to the current time, and ${\hat{a}}_{nb(k)}^{b}$ is the acceleration estimate of the previous moment. Equation (20) shows that $\bar{a}(k+1)$ is not only related to the current information ${\hat{a}}_{nb(k)}^{b}$, but also to the past information $\bar{a}(k)$.

Since the CS model assumes that the maneuvering acceleration at an arbitrary time obeys the modified Rayleigh distribution, the variance of body acceleration can be obtained as follows:

(21) $$\sigma_{a}^{2}=\{\begin{array}{c} \frac{4-\pi }{\pi }{\left[a_{\max}-\hat{a}(k-1)\right]}^{2}\begin{array}{c} 0 \end{array}<\hat{a}(k-1)<a_{\max}\\ \frac{4-\pi }{\pi }{\left[a_{\max}+\hat{a}(k-1)\right]}^{2}\begin{array}{c} a_{-\max}<\hat{a}(k-1)<0 \end{array} \end{array}.$$

As can be seen from Equations (13) and (18), the position vector r and the body velocity vector $v_{nb}^{b}$ are affected by the CS Singer acceleration model. Consequently, combining the 3D kinematic trajectory model in Section 2.1 and the CS Singer acceleration model in Section 2.2, the discrete system equations of kinematic trajectory can be derived as follows:

(22) $$\begin{array}{c} r(k+1)=r(k)+C_{n(k)}^{e}\{\Delta tC_{b(k)}^{n}v_{nb(k)}^{b}+\left[{\left(\alpha \Delta t-1+e^{-\alpha \Delta t}\right)/\alpha }^{2}\right]C_{b(k)}^{n}a_{nb(k)}^{b}\\ +\left[\frac{\Delta t^{2}}{2}-(\alpha \Delta t-1+e^{-\alpha \Delta t})/\alpha^{2}\right]C_{b(k)}^{n}\bar{a}(k)+C_{b(k)}^{n}w_{a}(k)+\frac{\Delta t^{3}}{6}C_{b(k)}^{n}j_{nb(k)}^{b}\} \end{array},$$

(23) $$\begin{array}{c} v_{nb(k+1)}^{b}=\left[I_{3\times 3}-\Delta t\left[\omega_{nb(k)}^{b}\times \right]+\frac{\Delta t^{2}}{2}{\left[\omega_{nb(k)}^{b}\times \right]}^{2}\right]v_{nb(k)}^{b}+\left[\left(1-e^{-\alpha \Delta t}\right)/\alpha \right]a_{nb(k)}^{b}\\ +\left[\Delta t-\left(1-e^{-\alpha \Delta t}\right)/\alpha \right]\bar{a}(k)+w_{a}(k)+\frac{\Delta t^{2}}{2}\left[v_{nb(k)}^{b}\times \right]{\dot{\omega }}_{nb(k)}^{b}+\frac{\Delta t^{2}}{2}j_{nb(k)}^{b} \end{array},$$

(24) $$a_{nb(k+1)}^{b}=e^{-\alpha \Delta t}a_{nb(k)}^{b}+(1-e^{-\alpha \Delta t})\bar{a}(k)+w_{a}(k).$$

The discrete system equations of the attitude and body angular rate are directly given as follows:

(25) $$\theta (k+1)=\theta (k)+\Delta tC_{3\times 3}\omega_{nb(k)}^{b}+\frac{\Delta t^{2}}{2}C_{3\times 3}{\dot{\omega }}_{nb}^{b},$$

(26) $$\omega_{nbx(k+1)}^{b}=e^{-\Delta t/T_{x}}\omega_{nbx(k)}^{b}+w_{\omega x},$$

(27) $$\omega_{nby(k+1)}^{b}=e^{-\Delta t/T_{y}}\omega_{nby(k)}^{b}+w_{\omega y},$$

(28) $$\omega_{nbz(k+1)}^{b}=e^{-\Delta t/T_{z}}\omega_{nbz(k)}^{b}+(1-e^{-\Delta t/T_{z}})\omega_{nbz(k-1)}^{b}+w_{\omega z}.$$

The discrete system equations of the systematic errors of three different low-cost IMUs are individually modeled as follows:

(29) $$b_{gi}(k+1)=b_{gi}(k)+w_{b_{gi}},$$

(30) $$b_{ai}(k+1)=b_{ai}(k)+w_{b_{ai}},$$

(31) $$s_{gi}(k+1)=s_{gi}(k)+w_{s_{gi}},$$

(32) $$s_{ai}(k+1)=s_{ai}(k)+w_{s_{ai}},$$

where i represents the sequence number of the IMU with i=1,2,3, $\Delta t=t_{k+1}-t_{k}$ is the time interval, $T_{x},T_{y},T_{z}$ are the time correlation coefficients of the first-order Markov model, $w_{\omega x},w_{\omega y},w_{\omega z}$ are the independent white noises for triaxial angular rates, And $w_{bg},w_{sg},w_{ba}$, and $w_{sa}$ are the white-noise vectors for the biases and scale factor errors of gyroscopes and accelerometers, while subscripts 1, 2, and 3 are sequence numbers of three different IMUs. $j_{nb}^{b}$ is treated as process noise for the position vector, velocity vector, and acceleration vector, ${\dot{\omega }}_{nb}^{b}$ is the angular acceleration as process noise for the velocity vector, acceleration vector, and attitude vector, and $C_{b}^{n}$ is the DCM. $C_{3\times 3}$ is the coefficient matrix as in Equation (14). $C_{n}^{e}$, the position cosine matrix, can be obtained through the position information as follows:

$$C_{n}^{e}=\left[\begin{array}{ccc} -\sin\lambda & -\sin\varphi \cos\lambda & \cos\varphi \cos\lambda \\ \cos\lambda & -\sin\varphi \sin\lambda & \cos\varphi \sin\lambda \\ 0 & \cos\varphi & \sin\varphi \end{array}\right],$$

wherein φ and λ are the latitude and longitude, respectively, calculated by the coordinate components of the position vector r in $S_{e}$.

### 3.3. The Measurement Model of IMU

**Figure 3:**
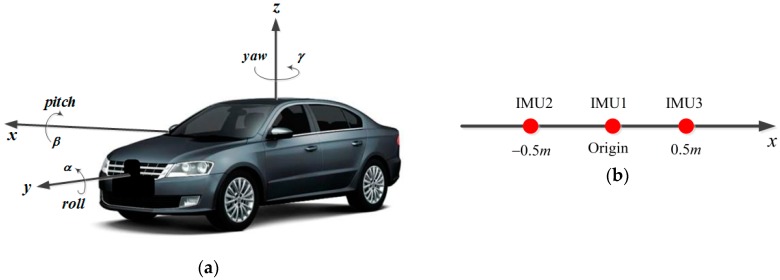
The structure diagram of multiple inertial measurement units (IMUs). (**a**) Vehicle’s general view; (**b**) partial enlarged details for internal structure.

Generally, the IMU raw outputs include specific forces from three orthogonal accelerometers and angular rates from three orthogonal gyroscopes. We should derive three groups of measurement equations because there are three IMUs in the integration system. Figure 3 shows the structure diagram of IMUs, where Figure 3a is the general view of the vehicle, and Figure 3b represents the partial enlarged details.

The measurement equations for low-cost IMUs could be simplified depending on specific needs [15,19]. Considering that IMUs cannot be located at the same point on the body, the measurements from different IMUs must be transformed to the same reference frame so as to perform the fusion algorithm [29]. Here, the central IMU is selected as the reference. Three groups of measurement equations for angular rate and specific force are respectively derived as shown below based on the particular structure.

(33) $$\omega_{ib-imu1}^{b}=(I+S_{g1})\omega_{nb}^{b}+b_{g1}+\Delta_{g1},$$

(34) $$\omega_{ib-imu2}^{b}=(I+S_{g2})\omega_{nb}^{b}+b_{g2}+\Delta_{g2},$$

(35) $$\omega_{ib-imu3}^{b}=(I+S_{g3})\omega_{nb}^{b}+b_{g3}+\Delta_{g3},$$

(36) $$f_{ib-imu1}^{b}=(I+S_{a1})(a_{nb}^{b}-C_{n}^{b}g^{n})+b_{a1}+\Delta_{a1},$$

(37) $$f_{ib-imu2}^{b}=(I+S_{a2})(a_{nb}^{b}-C_{n}^{b}g^{n})+\omega_{ib}^{b}\times (\omega_{ib}^{b}\times r_{2})+b_{a2}+\Delta_{a2},$$

(38) $$f_{ib-imu3}^{b}=(I+S_{a3})(a_{nb}^{b}-C_{n}^{b}g^{n})+\omega_{ib}^{b}\times (\omega_{ib}^{b}\times r_{3})+b_{a3}+\Delta_{a3},$$

where $g^{n}$ is the local gravity vector in $S_{n}$, $\omega_{nb}^{b},a_{nb}^{b}$ are the rotation rate and acceleration vectors of $S_{b}$ with respect to $S_{n}$, $S_{g},S_{a}$ are 3×3 scale factor error matrices for gyros and accelerometers, $b_{g},b_{a}$ have the same meanings as mentioned in Section 3.1, and $\Delta_{g,}\Delta_{a}$ are Gaussian white noises for the angular rate vector and specific force vector. The lever arm parameters of the remaining IMUs relative to the central one are $r_{2}={\left[-0.5,0,0\right]}^{T}$ and $r_{3}={\left[0.5,0,0\right]}^{T}$.

### 3.4. The Measurement Model of the GPS

In this study, not only do the raw observables of IMUs participate in measurement updates, but so do those of the GPS. The GPS, as another sensor distinct from the IMU but with equal status in the system, offers two types of observables (carrier phase and pseudo-range). However, only the pseudo-range is adopted to complete the specific navigation task. The pseudo-range observation equation is generally given as

(39) $$PR_{A}^{j}=\rho_{A}^{j}+C(\delta t_{A}-\delta t^{j})+d_{A-trop}^{j}+d_{A-ion}^{j}+\varepsilon_{PR_{A}^{j}},$$

where j=1,2,⋯,n denotes the j−th satellite, and $\rho_{A}^{j}=\sqrt{{\left(X^{j}-X\right)}^{2}+{\left(Y^{j}-Y\right)}^{2}+{\left(Z^{j}-Z\right)}^{2}}$ is the distance between satellite j and receiver A, where $\left(X^{j},Y^{j},Z^{j}\right)$ is the geocentric coordinate of satellite j, calculated by the relevant parameters provided in the satellite navigation message, c is the light speed, $\delta t_{A},\delta t^{j}$ are the clock errors of receiver and satellite, respectively, $d_{A-trop}^{j},d_{A-ion}^{j}$ are the tropospheric and ionospheric delays, and $\varepsilon_{PR_{A}^{j}}$ is the random noise.

## 4. The Implementation of the Kalman Filter

On the basis of the system model proposed above, the KF is structured straightforwardly. The state Equations (22)–(32) and measurement Equations (33)–(39) are separately generalized by the discrete nonlinear system and measurement models as shown below.

(40) $$X_{k+1}=f(X_{k})+B_{k}u_{k}+\Gamma_{k}W_{k},$$

(41) $$Z_{k}=h(X_{k})+\Delta_{k},$$

where $X_{k}$ is the state vector as determined in Section 3.1, $Z_{k}$ is the measurement vector as described in Section 3.3 and Section 3.4, f() and h() are nonlinear mathematical functions, and they are constructed from Equations (22)–(32) and Equations (33)–(39), respectively, $B_{k}$ and $\Gamma_{k}$ are coefficient matrices, $u_{k}$ is the system input, $u_{k}=\bar{a}(k)$, $\Delta_{k}$ is the measurement noise vector, and $W_{k}$ is the process noise vector including the jerk vector, the derivative of the angular rate vector, and so forth. Specifically, $B_{k}$, $\Gamma_{k}$, and $W_{k}$ are given as

Bk=[[Δt22−(αΔt−1+e−αΔt)/α2]⋅Cne⋅Cbn[Δt−(1−e−αΔt)/α]⋅I3×3(1−e−αΔt)⋅I3×3042×3]51×3,Γk(51×48)=[Cne⋅CbnΔt36Cne⋅Cbn03×3I3×3Δt22I3×3Δt2⋅[vnbb×]2I3×303×303×303×303×3Δt22C3×3012×39039×9I39×39],Wk=[(wa)T,(jnbb)T,(ω˙nbb)T,wωx,wωy,wωz,(wbg1)T,(wbg2)T,(wbg3)T,(wba1)T,(wba2)T,(wba3)T,(wsg1)T,(wsg2)T,(wsg3)T,(wsa1)T,(wsa2)T,(wsa3)T]T

As is well known, the Kalman filter performs the prediction of the state vector through two information update processes: time update and measurement update. Specifically, the one-step prediction and the variance propagation of the state vector proceed from epoch k to k+1 during the time update.

(42) $${\hat{X}}_{k+1/k}=f({\hat{X}}_{k})+B_{k}u_{k},$$

(43) $$P_{k+1/k}=\Phi_{k+1,k}P_{k}\Phi_{k+1,k}^{T}+\Gamma_{k}Q_{k}\Gamma_{k}^{T},$$

where $P_{k}$ is the mean square error matrix of the state vector, and $Q_{k}$ is the a priori variance–covariance matrix of the process noise vector. $\Phi_{k+1,k}$ is the Jacobian matrix of the nonlinear system model in Equation (40), with $\Phi_{\left[i,j\right]}=\frac{\partial f_{\left[i\right]}}{\partial X_{\left[j\right]}}$ (i,j represent the serial numbers; $f_{\left[i\right]}$ represents the *i*-th system equation; $X_{\left[j\right]}$ represents the *j*-th component of state vector X), as follows:

Φk+1,k=[I3×3Δt⋅Cne⋅Cbn∂rk+1∂ak03×303×303×3∂vk+1∂vk∂vk+1∂ak03×3∂vk+1∂ωk03×303×3e−αΔt⋅I3×303×3003×303×303×3∂θk+1∂θkΔt⋅C3×303×303×303×303×3∂ωk+1∂ωk015×36036×15I36×36],

with

∂rk+1∂ak=(αΔt−1+e−αΔt)⋅Cne⋅Cbn/α2, ∂vk+1∂vk=I3×3−Δt[ωnb(k)b×]+Δt22[ωnb(k)b×]2,

∂vk+1∂ak=(1−e−αΔt)⋅I3×3/α, and ∂vk+1∂ωk=Δt[vnb(k)b×]+Δt22Mk, where

$$M_{k}=\left[\begin{array}{ccc} v_{nby}^{b}\omega_{nby}^{b}+v_{nbz}^{b}\omega_{nbz}^{b} & -2v_{nbx}^{b}\omega_{nby}^{b}+v_{nby}^{b}\omega_{nbx}^{b} & -2v_{nbx}^{b}\omega_{nbz}^{b}+v_{nbz}^{b}\omega_{nbx}^{b}\\ v_{nbx}^{b}\omega_{nby}^{b}-2v_{nby}^{b}\omega_{nbx}^{b} & v_{nbx}^{b}\omega_{nbx}^{b}+v_{nbz}^{b}\omega_{nbz}^{b} & -2v_{nby}^{b}\omega_{nbz}^{b}+v_{nbz}^{b}\omega_{nby}^{b}\\ v_{nbx}^{b}\omega_{nbz}^{b}-2v_{nbz}^{b}\omega_{nbx}^{b} & v_{nby}^{b}\omega_{nbz}^{b}-2v_{nbz}^{b}\omega_{nby}^{b} & v_{nbx}^{b}\omega_{nbx}^{b}+v_{nby}^{b}\omega_{nby}^{b} \end{array}\right],$$

∂θk+1∂θk=[1−sinγωnbxbΔt+cosγωnbzbΔt0(sinγωnbxb−cosγωnbzb)(secP)2Δt1+(cosγωnbxb+sinγωnbzb)(tanP)Δt0(sinγωnbxb−cosγωnbzb)(secP)(tanP)Δt(cosγωnbxb+sinγωnbzb)(secP)Δt1],∂ωk+1∂ωk=[e−Δt/Tx000e−Δt/Ty000e−Δt/Tz].

Subsequently, the measurement update is performed as follows when the measurements at epoch k+1 are available, thus accomplishing the state estimation:

(44) $${\hat{X}}_{k+1}={\hat{X}}_{k+1/k}+K_{k+1}(Z_{k+1}-h({\hat{X}}_{k+1/k})),$$

(45) $$K_{k+1}=P_{k+1/k}H_{k+1}^{T}{\left(H_{k+1}P_{k+1/k}H_{k+1}^{T}+R_{k+1}\right)}^{-1},$$

(46) $$P_{k+1}=(I-K_{k+1}H_{k+1})P_{k+1/k},$$

where $K_{k}$ is the Kalman filter gain matrix, And $R_{k}$ is the a priori variance–covariance matrix of the measurement noise vector. $H_{k}$ is the Jacobian matrix of the nonlinear measurement model in Equation (41), with $H_{\left[i,j\right]}=\frac{\partial h_{\left[i\right]}}{\partial X_{\left[j\right]}}$ (i,j represent the serial numbers; $h_{\left[i\right]}$ represents the *i*-th measurement equation; $X_{\left[j\right]}$ represents the *j*-th component of state vector X), as follows:

Hk=[09×12∂ωib1(k)b∂ωk∂ωib2(k)b∂ωk∂ωib3(k)b∂ωkI9×909×9∂ωib1(k)b∂sg1(k)03×303×303×3∂ωib2(k)b∂sg2(k)03×303×303×3∂ωib3(k)b∂sg3(k)09×909×6∂fib1(k)b∂ak∂fib2(k)b∂ak∂fib3(k)b∂ak09×303×3∂fib2(k)b∂ωk∂fib3(k)b∂ωk09×9I9×909×9∂fib1(k)b∂sa1(k)03×303×303×3∂fib2(k)b∂sa2(k)03×303×303×3∂fib3(k)b∂sa3(k)∂PRk∂rk01×48⋮],

with

$$\frac{\partial \omega_{ib1(k)}^{b}}{\partial \omega_{k}}=I_{3\times 3}+S_{g1},\;\frac{\partial \omega_{ib2(k)}^{b}}{\partial \omega_{k}}=I_{3\times 3}+S_{g2},\;\frac{\partial \omega_{ib3(k)}^{b}}{\partial \omega_{k}}=I_{3\times 3}+S_{g3},\;\frac{\partial \omega_{ib1(k)}^{b}}{\partial s_{g1(k)}}=\frac{\partial \omega_{ib2(k)}^{b}}{\partial s_{g2(k)}}=\frac{\partial \omega_{ib3(k)}^{b}}{\partial s_{g3(k)}}=diag(\omega_{nbx}^{b},\omega_{nby}^{b},\omega_{nbz}^{b}),$$

$$\frac{\partial f_{ib1(k)}^{b}}{\partial a_{k}}=I_{3\times 3}+S_{a1},\;\frac{\partial f_{ib2(k)}^{b}}{\partial a_{k}}=I_{3\times 3}+S_{a2},\;\frac{\partial f_{ib3(k)}^{b}}{\partial a_{k}}=I_{3\times 3}+S_{a3},\;\frac{\partial f_{ib2(k)}^{b}}{\partial \omega_{k}}=\left[\begin{array}{ccc} \omega_{nbz}^{b}r_{2}(3)+\omega_{nby}^{b}r_{2}(2) & \omega_{nbx}^{b}r_{2}(2)-2\omega_{nby}^{b}r_{2}(1) & -2\omega_{nbz}^{b}r_{2}(1)+\omega_{nbx}^{b}r_{2}(3)\\ -2\omega_{nbx}^{b}r_{2}(2)+\omega_{nby}^{b}r_{2}(1) & \omega_{nbz}^{b}r_{2}(3)+\omega_{nbx}^{b}r_{2}(1) & \omega_{nby}^{b}r_{2}(3)-2\omega_{nbz}^{b}r_{2}(2)\\ \omega_{nbz}^{b}r_{2}(1)-2\omega_{nbx}^{b}r_{2}(3) & -2\omega_{nby}^{b}r_{2}(3)+\omega_{nbz}^{b}r_{2}(2) & \omega_{nbx}^{b}r_{2}(1)+\omega_{nby}^{b}r_{2}(2) \end{array}\right],\;\frac{\partial f_{ib3(k)}^{b}}{\partial \omega_{k}}=\left[\begin{array}{ccc} \omega_{nbz}^{b}r_{3}(3)+\omega_{nby}^{b}r_{3}(2) & \omega_{nbx}^{b}r_{3}(2)-2\omega_{nby}^{b}r_{3}(1) & -2\omega_{nbz}^{b}r_{3}(1)+\omega_{nbx}^{b}r_{3}(3)\\ -2\omega_{nbx}^{b}r_{3}(2)+\omega_{nby}^{b}r_{3}(1) & \omega_{nbz}^{b}r_{3}(3)+\omega_{nbx}^{b}r_{3}(1) & \omega_{nby}^{b}r_{3}(3)-2\omega_{nbz}^{b}r_{3}(2)\\ \omega_{nbz}^{b}r_{3}(1)-2\omega_{nbx}^{b}r_{3}(3) & -2\omega_{nby}^{b}r_{3}(3)+\omega_{nbz}^{b}r_{3}(2) & \omega_{nbx}^{b}r_{3}(1)+\omega_{nby}^{b}r_{3}(2) \end{array}\right],$$

$\frac{\partial f_{ib1(k)}^{b}}{\partial s_{a1(k)}}=\frac{\partial f_{ib2(k)}^{b}}{\partial s_{a2(k)}}=\frac{\partial f_{ib3(k)}^{b}}{\partial s_{a3(k)}}=diag(N_{x},N_{y},N_{z})$, wherein, ${\left[N_{x},N_{y},N_{z}\right]}^{T}=(a_{nb}^{b}-C_{n}^{b}g^{n})$,

∂PRk∂rk=[(X−Xj)kρAj(k)(Y−Yj)kρA(k)j(Z−Zj)kρA(k)j].

## 5. Road Test and Results

**Figure 4:**
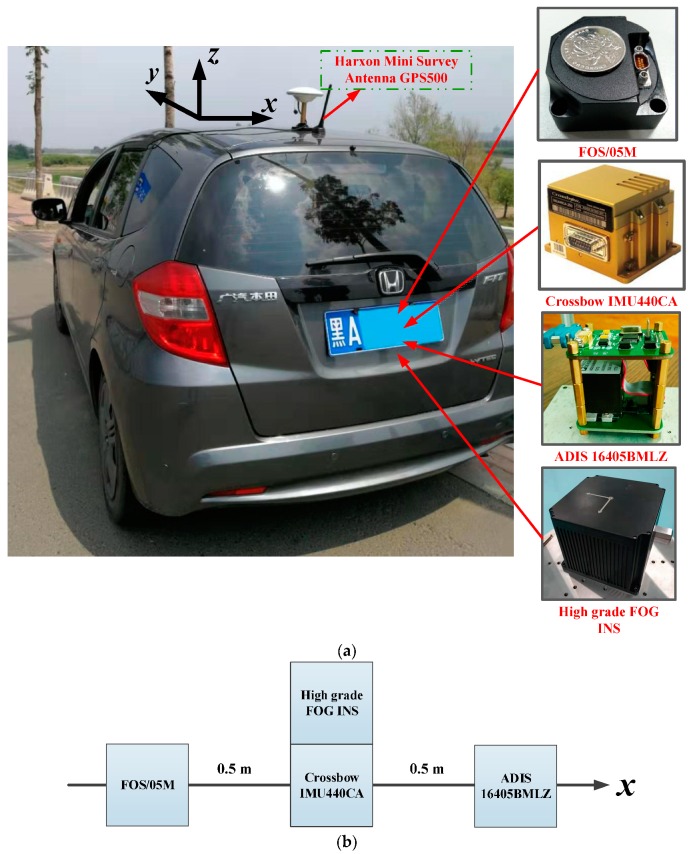
The equipment installation diagram for the road test. (**a**) The test vehicle and sensors; (**b**) internal installation diagram.

**Figure 5:**
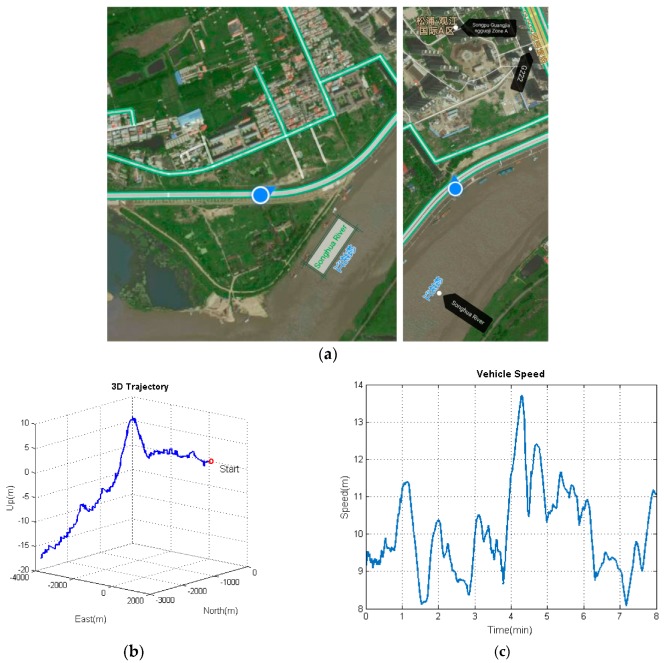
The trajectory and velocity of the test vehicle. (**a**) Trajectory capture map; (**b**) three-dimensional (3D) trajectory; (**c**) velocity profile.

The proposed algorithm model under the unconventional integration strategy was adopted to process the navigation data from multiple low-cost IMUs and a GPS-integrated system on a land vehicle. Several road tests were performed by our ground-based vehicle navigation system with a Harxon Mini Survey Antenna GPS500 and three IMUs (FOS/05M, ADIS16405BMLZ, and Crossbow IMU440CA), as shown in Figure 4. Table 1 shows the performance parameters of the three IMUs. The experimental results are given in this section from one of our road tests. The used dataset was collected in Harbin, China, from which an 8-min data fragment of the data source was selected for demonstration purposes. Figure 5a reveals the environment where the measurements were made. As can be seen from Figure 5a, the chosen test environment was an urban highway, with the Songhua River and buildings nearby. Figure 5b,c depict the trajectory and velocity profile of the test vehicle.

In order to obtain the ground truth as the reference, we equipped a high-grade Fiber-Optic Gyroscope (FOG) inertial navigation system (INS) (gyroscope: constant drift less than 0.01°/h, random noise less than 0.001°/h; accelerometer: constant bias less than 100 μg, random noise less than 10 μg) developed by our research group on the test vehicle, as shown in Figure 4a. Hence, the benchmarks for attitude, velocity, and position could be provided by fusing the measurements of the high-grade INS and the GPS.

### 5.1. Further Insights into the Unconventional Integration Mechanism

**Figure 6:**
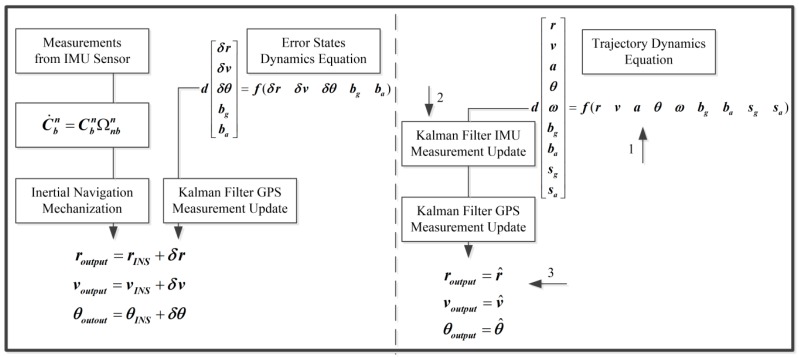
Comparison with conventional global positioning system (GPS)-aided IMU integration mechanism.

The distinctions between the conventional integration mechanism (left side) and the unconventional counterpart (right side) are shown in Figure 6: (1) the embedment of the prediction of the kinematic states, (2) the introduction of IMU measurements, and (3) the navigation parameters directly used in the state vector.

The acceleration vector and angular rate vector are only regarded as inputs of the mechanization in the conventional error state-based Kalman filter, while the realistic measurements of the IMU directly participate in the measurement update process in the unconventional Kalman filter. In principle, the Kalman filter is equivalent to a sequential least square with a time-variant state vector and the process noises [30]. That is to say, the system model is composed of a group of virtual measurements for the state vector and reflects the connections between the state vectors from epoch to epoch. These extra virtual measurements mean that the measurement redundancies in the unconventional Kalman filter for the body acceleration vector $a_{nb}^{b}$ and the body angular rate vector $\omega_{nb}^{b}$ are evidently better than those in the conventional Kalman filter. Therefore, the accuracy of $a_{nb}^{b}$ and $\omega_{nb}^{b}$ in the unconventional KF for multiple low-cost IMUs and a GPS-integrated system will be undoubtedly improved because they are not only predicted but also measured. The prediction for $a_{nb}^{b}$ and $\omega_{nb}^{b}$ in the system model can be used as a rigorous reference to check on the performance of the IMU without increasing the complexity of the filtering structure. Furthermore, the system equations of the unconventional KF can act as the dynamic constraints for the navigation parameters, for example, assuming that $v_{nbz}^{b}=0$ and/or $v_{nbx}^{b}=0$.

Generally speaking, the most significant feature of the unconventional integration mechanism lies in the improvement of the overall measurement redundancy of the system through the system model based on the 3D kinematic trajectory model.

**Figure 7:**
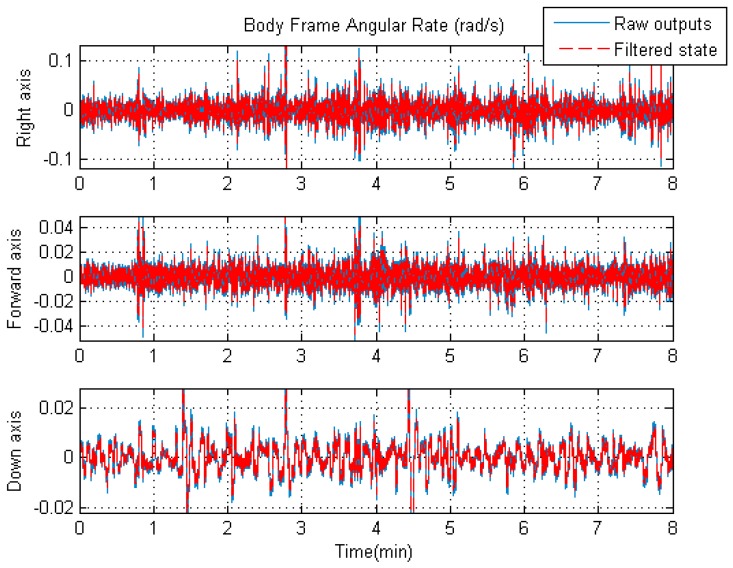
The raw angular rate of the central IMU and the filtered angular rate.

**Figure 8:**
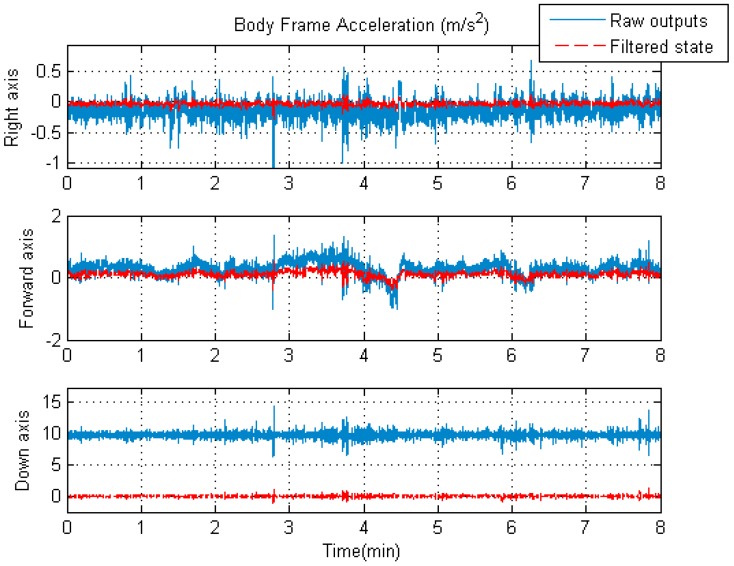
The raw specific force of the central IMU and the filtered acceleration.

As discussed above, it is expected that the accuracy of the navigation solutions shall be improved by using the rigorous trajectory model as the system model in the unconventional KF. As the components of the state vector, the acceleration and angular rate vectors in the body frame ($S_{b}$) will also profit from the unconventional KF time updates for the same reason. For better visual effects, Figure 7 and Figure 8 show the comparisons between the raw IMU outputs and filtered signals.

As can be seen from Figure 7, the overall variation of the IMU raw angular rates is slightly larger than that of the filtered angular rates. However, it is apparent from Figure 8 that the overall fluctuation of the filtered accelerations is significantly lower than that of the IMU raw specific forces. The improvement is due to the introduction of the novel system model in the unconventional Kalman filter. It is worth mentioning that the raw output of the accelerometer in the vertical direction includes acceleration due to gravity, while the corresponding filtered acceleration does not. That is why there is a large difference (about 10 ${m/s}^{2}$) between the raw and filtered acceleration values along the vertical axis in Figure 8; here, we only compare the fluctuating ranges of the two curves.

The comparison results from Figure 7 and Figure 8 confirm the fact that the influences of the IMU measurement noises on the final navigation solutions are effectively mitigated due to the participation of the IMU outputs in the KF measurement updates.

### 5.2. Verification of the Proposed Algorithm Model under the Unconventional Integration Strategy

**Figure 9:**
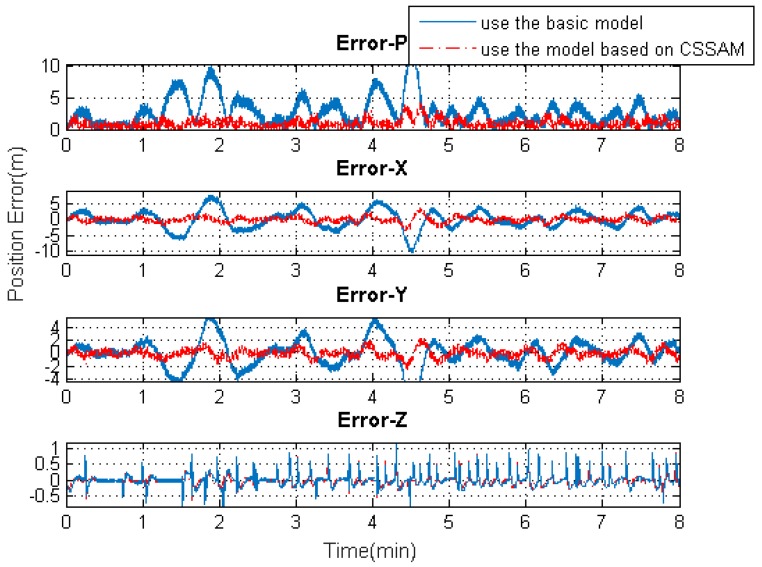
The 3D position solution accuracy comparisons using the basic model or the model based on the “current” statistical Singer acceleration model (CSSAM).

**Figure 10:**
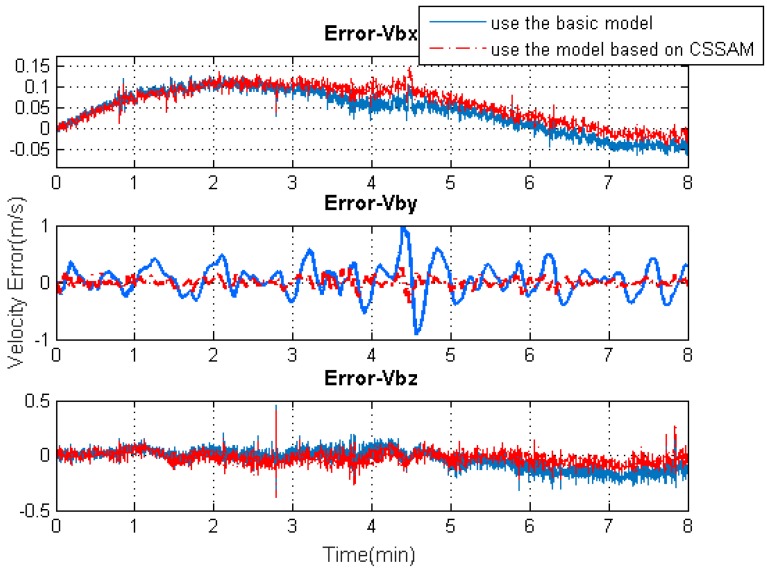
The 3D velocity solution accuracy comparisons using the basic model or the model based on the CSSAM.

**Figure 11:**
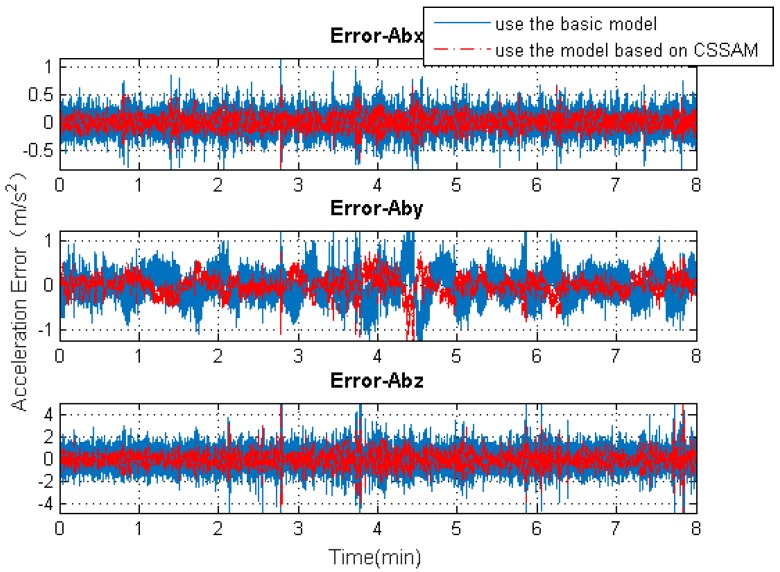
The 3D acceleration solution accuracy comparisons using the basic model or the model based on the CSSAM.

**Figure 12:**
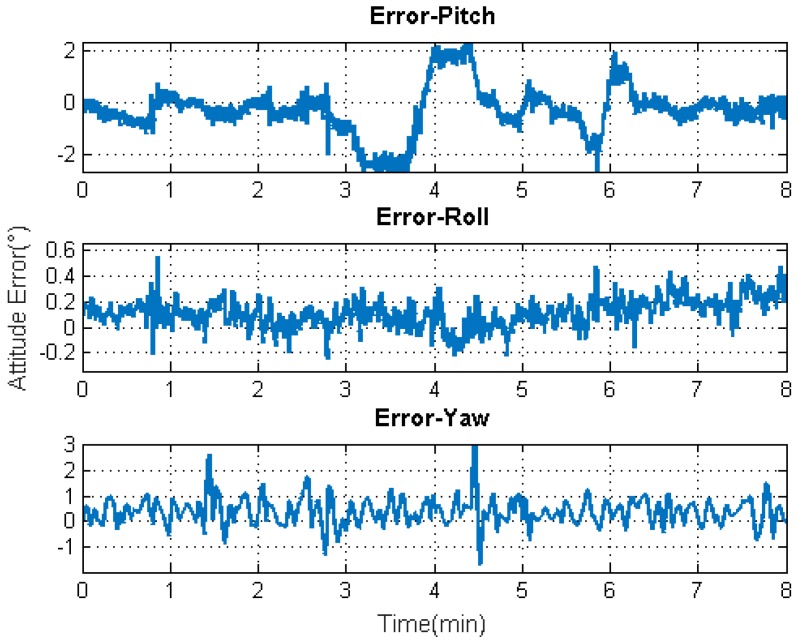
Attitude solution accuracy.

Figure 9, Figure 10, Figure 11 and Figure 12 exhibit the solution accuracies for the kinematic trajectory parameters and attitude using the proposed algorithm model under the unconventional integration strategy.

Figure 9 shows the solution accuracy comparisons for 3D position using the basic 3D kinematic trajectory model or the practical model based on the “current” statistical Singer acceleration model (CSSAM). The overall 3D position accuracy using the proposed algorithm model is under 5 m, which is a great improvement compared with the accuracy (10 m) using the basic one. Figure 10 shows that the estimation errors for the velocity state vector in the three axial directions of the body frame using the proposed algorithm model are within −0.05∼0.15 m/s, ±0.2 m/s, and ±0.2 m/s, respectively. The velocity error in the direction of travel is obviously reduced compared with that using the basic model. Figure 11 shows that the estimation errors for acceleration state vector in the three axial directions of the body frame are within ±0.5 $m/s^{2}$, ±1 $m/s^{2}$, and ±2 $m/s^{2}$, respectively. As can be seen, the accuracy of the acceleration estimation is apparently improved by using the proposed algorithm model. As shown in Figure 12, the accuracies for attitude (pitch, roll, and heading) are within ±2°, −0.2∼0.6°, and −1∼3°, respectively. It is to be observed that the pitch angle has an apparent fluctuation from 3–4.5 min. The trajectory curve in Figure 5b illustrates that the vehicle experienced ups and downs in the test duration, which could affect the estimation of attitude angles (especially for pitch angle). It also indicates that the attitude estimation model still needs improvement so that it can be applied to more complex motion forms.

Apparently, the navigation parameters during the 8-min vehicle motion were estimated within acceptable ranges by adopting the proposed algorithm model under the unconventional integration strategy. Furthermore, the reason why the 3D position error using the basic trajectory model did not diverge is that the output of the central accelerometer with real-time calibration was utilized to regulate the acceleration estimation error when significant acceleration maneuvers occurred.

### 5.3. IMU Systematic Error Estimation

**Figure 13:**
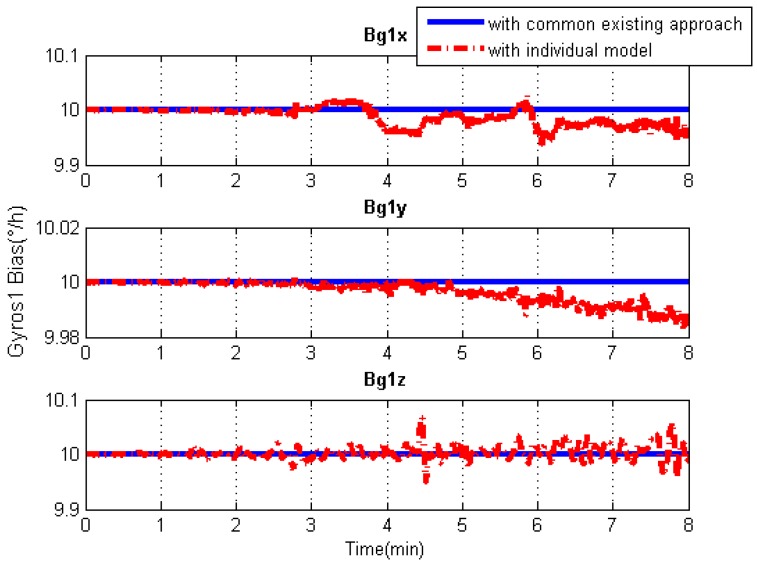
Gyroscope bias solution comparisons of the central IMU using the common existing approach or the individual modeling method.

**Figure 14:**
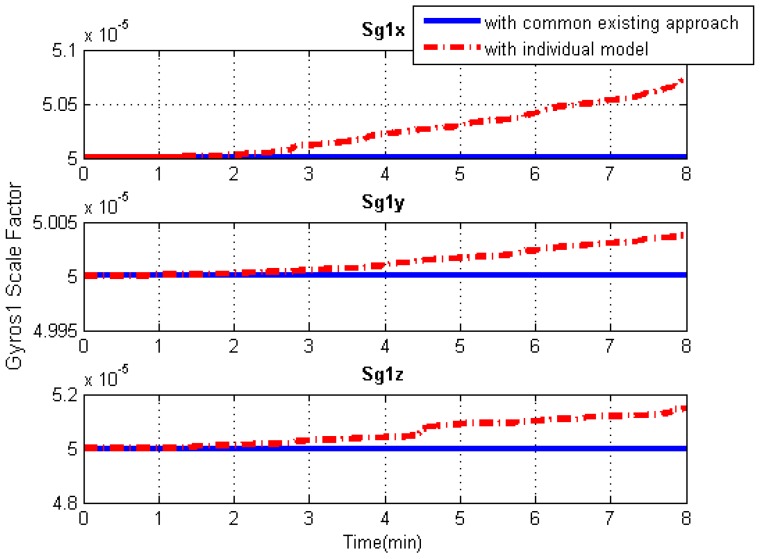
Gyroscope scale factor solution comparisons of the central IMU using the common existing approach or the individual modeling method.

**Figure 15:**
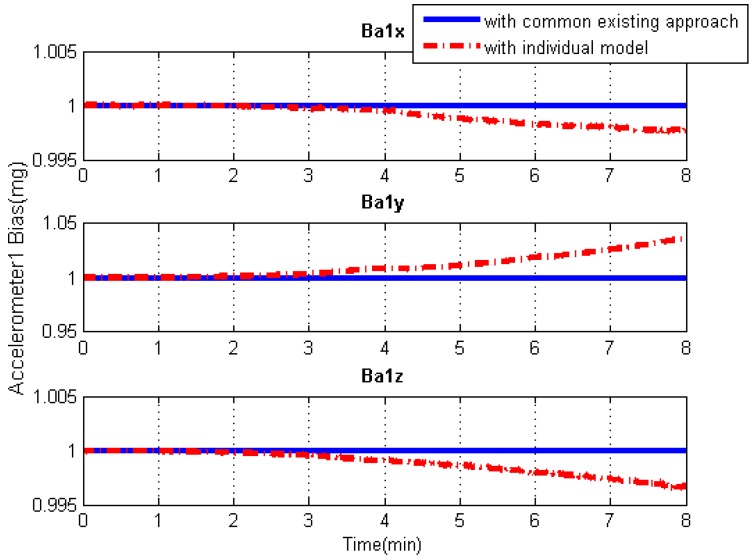
Accelerometer bias solution comparisons of the central IMU using the common existing approach or the individual modeling method.

**Figure 16:**
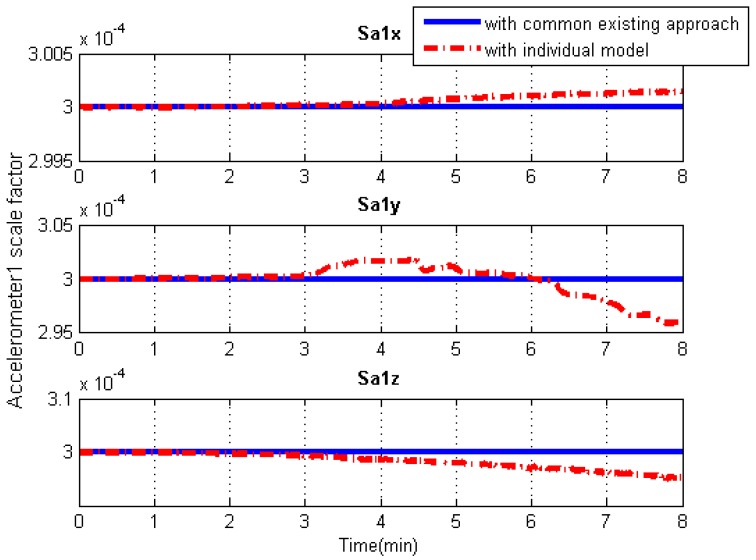
Accelerometer scale factor solution comparisons of the central IMU using the common existing approach or the individual modeling method.

Since the systematic errors of these IMU arrays, i.e., biases and scale factor errors of gyroscopes and accelerometers, are individually modeled in Kalman filtering, the systematic error estimations of each IMU can be obtained separately, as illustrated in Figure 13, Figure 14, Figure 15 and Figure 16. As an example, this manuscript only shows the systematic errors of the central IMU due to space limitations. The common existing approach under the conventional inertial navigation mechanization adopts a set of common shared errors for all IMUs and, sometimes, these error parameters are from initial calibration results or technical specifications [7,28]. However, in fact, the a priori error model defined for a static low-cost MEMS inertial sensor needs to be checked and compensated for in the dynamic working environment as the vibration on a low-cost IMU might cause significant changes in its scale factors and noise level compared to those in the static case [31].

As can be seen from Figure 13, Figure 14, Figure 15 and Figure 16, in contrast to the a priori constant systematic error with the common existing approach, the systematic error estimations with the individual modeling method vary with time, which conforms better with the real situation. In other words, the individual modeling method under the unconventional integration strategy can adjust the systematic error estimations of each IMU according to the real-time measurements from each IMU. This technique can firstly verify if those IMUs really share the same systematic errors quantitatively, even if they are the common errors physically; then, they can be modeled either separately or combined, laying the groundwork for future research such as auto calibration and fault detection. While applying the individual modeling method for the IMU array, in spite of its characteristic properties, one must confront the following problem: high computation load caused by high-rate measurement updates in the Kalman filter, e.g., with IMUs whose measurement rate may be at 100 Hz. Although the modern computation capability is considerably improved, one cannot stand such a high measurement update rate, especially one so unreasonable, which renders it useless in real time. How to appropriately reduce the high-rate KF measurement updates without compromising the valuable information embedded in the high-rate measurements will become a topic for further study.

## 6. Conclusions

This research fused the information from one GPS receiver and three low-cost IMUs by applying an unconventional multi-sensor integration strategy. The enhanced and improved parts involved establishing a more practical 3D kinematic trajectory model based on the “current” statistical Singer acceleration model as the core of the system model, and individually modeling the measurements and systematic errors of these IMU arrays in Kalman filtering. The processing results of the experimental data demonstrated the success of the proposed algorithm model under the unconventional integration strategy with satisfactory solution performance and reliability. Future work will involve developing a more precise fusion algorithm by using the carrier phase information from the GPS.

## Figures and Tables

**Table 1:** Performance parameters of the inertial measurement units (IMUs). USD—United States dollars.

	Specifications of Gyroscope	Specifications of Accelerometer	Cost (USD)
FOS/05M	In-run bias stability ≤7.2°/h Angular random walk ≤5.5°/$\sqrt{\mathrm{hr}}$	In-run bias stability ≤1.0 mg Velocity random walk ≤1.0 m/s/$\sqrt{\mathrm{hr}}$	1500
ADIS16405BMLZ	In-run bias stability ≤25.2°/h Angular random walk ≤2.0°/$\sqrt{\mathrm{hr}}$	In-run bias stability ≤0.2 mg Velocity random walk ≤0.2 m/s/$\sqrt{\mathrm{hr}}$	600
Crossbow IMU440CA	In-run bias stability ≤10.0°/h Angular random walk ≤4.5°/$\sqrt{\mathrm{hr}}$	In-run bias stability ≤1.0 mg Velocity random walk ≤1.0 m/s/$\sqrt{\mathrm{hr}}$	1200
